# *Conus* Envenomation of Humans: In Fact and Fiction

**DOI:** 10.3390/toxins11010010

**Published:** 2018-12-27

**Authors:** Alan J. Kohn

**Affiliations:** Department of Biology, Box 351800, University of Washington, Seattle, WA 98195, USA; kohn@uw.edu; Tel.: +1-206-616-4383

**Keywords:** venomous marine snails, human injuries, temporal envenomation patterns

## Abstract

Prominent hallmarks of the widely distributed, mainly tropical marine snail genus *Conus* are: (1) its unusually high species diversity; it is the largest genus of animals in the sea, with more than 800 recognized species; and (2) its specialized feeding behavior of overcoming prey by injection with potent neurotoxic, paralytic venoms, and swallowing the victim whole. Including the first report of a human fatality from a *Conus* sting nearly 350 years ago, at least 141 human envenomations have been recorded, of which 36 were fatal. Most *Conus* species are quite specialized predators that can be classified in one of three major feeding guilds: they prey exclusively or nearly so on worms, primarily polychaete annelids, other gastropods, sometimes including other *Conus* species, or fishes. These differences are shown to relate to the severity of human envenomations, with the danger increasing generally in the order listed above and a strong likelihood that all of the known human fatalities may be attributable solely to the single piscivorous species *C. geographus*.

## 1. Introduction

A database of reported human injuries and fatalities due to envenomation by marine gastropod molluscs of the genus *Conus*, maintained over the past 62 years, now lists 141 cases reported during the period 1670–2017. This account briefly summarizes their dates, geographic locations, outcomes, and identifications of perpetrator species, drawing on the author’s three prior reports on this topic [1,2,3]. It also compiles and analyzes previously overlooked data, particularly temporal trends in frequency and severity of human envenomations, ecological roles in nature of perpetrator species and how these affect human responses to stings, and cases reported subsequent to the publications cited above.

The *Conus* species responsible for about 95% of known human envenomations have been identified with a reasonable degree of certainty [3]. The other cases, in which the perpetrator was not clearly observed or identified, are designated by question marks in the species column of SM1 in the website cited above.

In nature, with few exceptions, *Conus* species are specialized predators on a narrow range of prey taxa, typically annelid worms, other gastropods, or fishes [4,5]. Across this vast genus, with more than 800 valid extant species currently recognized [6], the prey they consume in nature varies considerably among species. Those known to have stung humans with the most adverse physiological effects are primarily piscivorous, and they are only recently beginning to be subjected to exploration of the relationships between the natural prey of the particular *Conus* species, the specific neurotoxic peptides that comprise the injected venom, and the type and severity of outcomes experienced by human victims [7].

Finally, reported cases of human envenomation by *Conus* have inspired a few authors as the bases for fictional murder mysteries. These are briefly reviewed following the accounts of actual cases reported subsequently to the most recent prior compilation [3].

## 2. Results

### 2.1. Temporal Trends of Fatal and Non-Fatal Reported Cases of *Conus* Envenomations of Humans, 1670–2017

**Figure 1:**
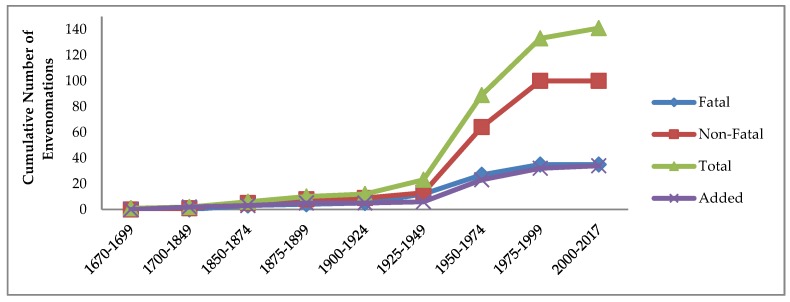
Temporal trends indicated by cumulative numbers and severity of reported human injuries from *Conus* envenomations, 1670–2017. Triangles and green line: all known cases; Squares and red line: non-fatal cases; Diamonds and blue line: fatalities. The X’s and purple line indicate the number of different *Conus* species responsible for human envenomations.

Prior studies have not explicitly examined the patterns over time of severity of human responses to reported *Conus* stings. Table 1 and Figure 1 show the trends in numbers and severity of the 141 known human injuries reported from the 34 *Conus* species known to be responsible, dating from the initial account of ca. 1670 [8] through to 2017. The IJCPT website distinguishes three levels of severity of *Conus* stings, fatal (designated “A”), serious symptoms followed by complete recovery (designated “B”), and minor effects only (comparable to a bee sting and designated “C”). For simplicity and clarity of the graphs in this paper, these are condensed into two categories, fatal (F, for outcome A), and non-fatal (NF, combining the two survivor outcomes B and C) (Figure 1).

The marked change in slopes, especially of the red and green lines in Figure 1, strongly suggest that starting from the second quarter of the 20th century, higher proportions of non-fatal cases were reported in news media than in the previous three centuries, followed by that trend leveling off in the 21st century. All points in Figure 1 from the first case through 1925–1949 indicate fatality rates of 42–100%. Thereafter, 70–75% of all reported cases resulted in recovery (Table 1). The lower cumulative number and proportion of cases resulting in loss of life during the later 20th century and into the 21st may be attributed mainly to increasing availability of medical treatment and of media in formerly more remote tropical regions reporting less serious cases.

### 2.2. Relation of Natural History of *Conus* Species to Severity of their Stings of Humans

Differences in the natural prey organisms of *Conus* species are strongly correlated both with the likelihood that a human will be stung and with the severity of the effects of envenomation. As noted briefly above, almost all *Conus* species whose diets in nature have been studied belong to one of three predatory feeding guilds, often referred to as worm-hunters, mollusc-hunters, and fish-hunters [9]. Probably more than two-thirds of all species are specialist predators on segmented worms of the phylum Annelida. In addition to including most species in the genus, the vermivorous guild is evolutionarily the oldest; its fossils first appear in the early Eocene epoch, about 57 million years before present (mybp). The other feeding guilds evolved from it. The ages of origin of these clades are uncertain, but recent molecular phylogenetic analysis of the Conidae [10] and systematic classification of Conoidea [5] indicate that the fossil record of the extant piscivorous subgenus *Pionoconus* first appeared in the Upper Oligocene (about 30 mybp). In contrast, the fossil record suggests that the origin of molluscivory in *Conus* is much more recent. The oldest fossils of an extant molluscivorous subgenus appear to be those of *C. kanayai* [11], from the latest Miocene (about 5–7 mybp). Shuto [11] originally placed *C. kanayai* in the genus *Conolithus.* Tucker and Tenorio [5] later correctly assigned it to *Cylinder*, which is now classified as a subgenus of *Conus* [10].

Like many other gastropods, *Conus* of all species that have been tested recognize the presence of appropriate prey at a distance from chemical signals released in the water [12,13]. They move toward the prey, apparently following the concentration gradient of the chemoattractants, and extend the tubular proboscis toward the prey. Upon making contact, the proboscis musculature injects a complex, species-specific venom pumped through a disposable, hollow, hypodermic needle-like radular tooth (Figures 1 and 2 in Ref. [3]) from the proboscis into the prey, which is rapidly immobilized and swallowed whole [14]. Several recent reviews survey the structure, target receptors, and functions of these highly neurotoxic compounds, primarily small peptides, as well as their increasing therapeutic roles [15,16,17].

### 2.3. Piscivorous *Conus* Species

**Figure 2:**
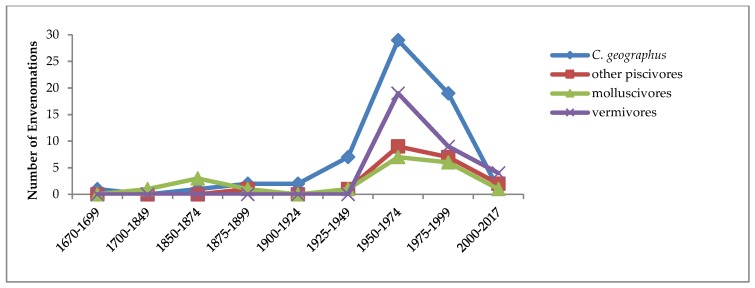
Temporal trends in the numbers of human injuries caused by species of *Conus* that are specialized predators on fishes, other gastropod molluscs, or worms, 1670–2017. Data for *C. geographus* are plotted separately (blue line and diamonds) because it has been responsible for more than half of all reported human injuries, as well as most, if not all, known human fatalities.

As Figure 2 shows, more than half of all known human envenomations (57%) have resulted from stings by the single large, widely distributed, exclusively piscivorous species *Conus geographus*. It is also quite likely that this species has caused all of the human fatalities, although species identity of the perpetrators is still uncertain in a few cases (about 8; see SM1). They have been ascribed in the literature to other *Conus* species, primarily the large, exclusively molluscivorous *C. textile,* but none of these has been adequately verified (see SM7). No fatalities have been reported from the nine other piscivorous species known to have stung humans (*n* = 20 cases; SM3).

Why *Conus geographus* is by far the most dangerous species to humans has not been thoroughly studied, particularly with respect to how the *Conus* becomes aware of the presence of an adversary worthy of attack. Chemical attractant signals are almost certainly released into the water by appropriate prey of molluscivorous and vermivorous, as well as piscivorous *Conus* species, but in contrast to substantial research on the effective toxic conopeptides [15,16,17], the nature of these initial attractants remain largely unknown [12,13,18].

The temporal pattern of reported human attacks by other piscivorous species (the red curve in Figure 2) is similar to that of *C. geographus.* These signal molecules likely differ among the three main feeding guilds of *Conus* species, but experimental data on food preference derive mainly from studies of vermivores (e.g., Ref. [18]), and I am not aware of any recent studies. The attractant molecules released by fishes are possibly more general attributes of vertebrates.

Fish-hunting *Conus* species typically share a feeding strategy that involves a very long, often curved radular tooth (often about 13% of shell length), with wide interspecific variation in prominence of blade, barbs and serrations (Figure 8 in Ref. [19]; see also Figure 13.2 in Ref. [17]). Prey is typically subdued by a single injection of venom in each feeding event. The tooth remains attached to the prey by its arrangement of barbs, and to the predator’s proboscis by contraction of a sphincter muscle in the proboscis in front of the basal spur when the tooth is injected. The tooth thus functions as a harpoon as well as a hypodermic needle, holding the prey until it gradually becomes paralyzed and can be completely engulfed.

Body size is also an important factor in the danger of *C. geographus* envenomation of humans. It is the largest extant piscivorous species in the genus, reaching a shell length of 166 mm [4]; very few piscivorous *Conus* species attain a shell length in excess of 100 mm. Body size, usually measured as shell length, is strongly correlated with severity of the injuries that *C. geographus* can inflict on people (Figures 4 and 5 in Ref. [3]). It is highly likely, but to my knowledge undocumented, that the larger the animal, the more venom it can inject in a single sting. The shells of the eight *C. geographus* individuals that are known to have killed people and whose shells have been measured ranged 80–135 mm in length (mean = 103 mm), while those that inflicted non-fatal stings ranged 50–125 mm in length (mean = 86 mm) (SM1). All of the shells of piscivores other than *C. geographus* that are known to have stung people (22 individuals of 11 species) were smaller; those whose shell lengths are known ranged from 24 to 55 mm (SM3).

One case (No. 133; SM1, SM3) involving the piscivorous species *Conus fulmen* deserves special mention because it involves the only known intentional human injection of *Conus* venom. *C. fulmen* occurs from Taiwan to the main Japanese islands, and by 1977, only one mild human injury from a sting had been reported (Case No. 78). Professor Shigeo Yoshiba, then of the Jikei University School of Medicine in Tokyo, decided to assess its danger and toxicity by injecting himself with its venom (Case No. 133). In addition, he carried out similar experiments on other mammals (mice and rabbits), two amphibians, five fishes, and members of five invertebrate phyla as well [20].

After injecting crude venom from *Conus fulmen* intracutaneously at a concentration of 0.0014 mg/kg into his forearm, Yoshiba stated that “no neurological or functional disturbance appeared; only local findings such as pain, redness, ischemia, edema, and itching appeared and lasted for 3 days” (p. 112 in Ref. [20]).

### 2.4. Molluscivorous *Conus* Species

There are about as many extant specialist molluscivorous *Conus* species as there are predators on fishes. However, in general the venom of the former guild is less toxic to humans, and members of only six different species are reported to have stung people (SM4). The piscivorous clades have a long evolutionary history of adapting their chemical weapons to overcoming vertebrate prey with a single dose of a formidable cocktail of numerous neurotoxic peptides, typically injected by a strongly barbed hypodermic radular tooth (Figure 8 in Ref. [19]) that also functions to catch and hold the prey until it can be engulfed by the rhynchocoel (= proboscis sheath or rostrum) (Figure 5.6 in Ref. [7]).

Successful predation on molluscs, most of which have strong protective shells that they can withdraw their bodies into when an enemy threatens or has already stung once, imposes different challenges. Almost all molluscan prey of *Conus* are other gastropods, and while a few species prey partly on shell-less nudibranchs, most molluscivorous *Conus* specialize on other shelled prosobranchiate gastropods, even including other *Conus* species in some cases.

These prey species often respond to being stung by withdrawing quickly (for a gastropod) into the shell, thus avoiding capture, perhaps until danger passes. However, the radular tooth form in molluscivorous *Conus* species follows different functions. They are typically slender, very elongated, and armed with a single moderately strong barb. They also have a narrower, more conical base without a spur, features that facilitate leaving the tooth embedded in the victim when feeding, in contrast to teeth of the other two feeding guilds (Figures 1–7 in Ref. [19]). Molluscivorous *Conus* species also tend to be patient and prepared to sting their injured victim again (and sometimes again) when it attempts to emerge from its shell.

Schoenberg [21] first reported the injection of multiple radular teeth by a molluscivorous *Conus* in the same feeding episode, in *C. textile* in Hawaii. To my knowledge, this staged encounter established the record number of radular teeth injected in a single predation episode. In this case the prey was another molluscivorous neogastropod, *Harpa amouretta.* A common defense of *Harpa* spp. to attempted predation is autotomy of the posterior portion of the foot. In this case, the *H. amouretta* did so upon recognizing the presence of the *C. textile*, but before the latter was able to inject a tooth into it. Subsequently, it injected five teeth into the *Harpa*’s shell aperture. These were not immediately lethal, the *H. amouretta* continued to wave its siphon, and the *C. textile* proceeded to inject 12 more radular teeth into its victim. Then, as the author described it, “When the *amouretta* stopped moving, Sir Textile began his meal. Fifteen minutes later, only an empty shell remained and the *textile* lazily slid down into the sand for a nap” (p. 4 in Ref. [20]).

More recently, Yoshiba studied the feeding process in *Conus bandanus* [22], and in greater detail in nearly 200 feeding events by a captive *C. textile* over five years in Japan. He showed that each feeding event averaged the injection of three radular teeth, with a maximum of six [23].

Kohn followed and videotaped an individual of *Conus victoriae*, a species closely related to *C. textile,* preying on the buccinid gastropod *Cantharus erythrostomus*, in Western Australia [24]. It successfully injected four teeth, after missing the target with one, over a period of about 29 min. It then completed swallowing the prey’s body within the next 6.5 h, after the venom had weakened the bond between the shell and columellar muscle. 

Although Prator et al. considered the reason why molluscivorous species inject multiple venom-laden teeth in one feeding event to be unclear [9], this strategy has likely been selected because the first one or two injections often miss the target or fail to immobilize the prey, which may extend from its shell and move away. Succeeding injections result in the victim remaining within its shell, increasingly unable to extend and more easily extracted intact from the shell and engulfed by the *Conus* due to weakened shell–muscle adhesion.

The habit of molluscivorous *Conus* injecting more than one tooth while overcoming a single prey organism is also exemplified by the only known case of a human having been stung more than once in the same attack episode. In this case (No. 128; SM4) a *C. bandanus* 48 mm long stung the victim on the same finger three times in succession, near a remote, uninhabited island in the Solomons. The most severe symptoms included an immediate burning sensation on the hand, blurred vision, lightheadedness, and chest pain with some difficulty breathing, which ameliorated after 24 h, headache that persisted for two weeks, and numbness and stiffness of the finger for about one week. The victim utilized the only medications available, meat tenderizer topically and strong antihistamine tablets, and recovered fully.

### 2.5. Vermivorous *Conus* Species

Annelid worms in the class Polychaeta, segmented relatives of earthworms, are the predominant prey of the numerically predominant vermivorous *Conus* species. Some annelids move freely about or burrow in sandy or muddy sediments, while others secrete and dwell in tubes that they construct of organic matter or calcium carbonate. The feeding biology of vermivorous *Conus* species more closely resembles that of piscivores than molluscivores. Like the former, in each feeding event they inject a single radular tooth containing enough venom to overcome the prey, which is often also harpooned, then engulfed and swallowed whole.

Although the majority of *Conus* species are vermivorous, reported stings of humans by them are both infrequent and limited to mild effects. Only 30 stings of humans have been reported, by 20 different vermivorous species (SM1, SM5). Two-thirds of these had only mild effects on victims, usually no more serious than a bee sting, and none have been life-threatening. The earliest reported cases occurred in 1955, and the most recent, in July 2017, from *C. regius* in Key West, Florida. In Figure 2, the purple curve shows that from the mid-20th century, the temporal pattern of stings by vermivorous species closely resembles those of the other *Conus* feeding guilds, suggesting that it reflects increased media interest in even mild molluscogenic human injuries.

### 2.6. Are *Conus* Stings of Humans Offensive or Defensive?

While offensive use of envenomation of prey by *Conus* has been well documented for nearly a century [25], only recently has evidence in favor of defensive use against perceived natural enemies gained support, importantly in the work of Dutertre and colleagues. These studies, summarized in [17,26], have shown that different conopeptides, that originate in different regions of the venom gland are deployed in the two different situations.

### 2.7. Positive Interaction of Humans and Conotoxins

While cone snails have killed more than 30 people and injured more than a hundred others, many conotoxins are under intensive study for applications in medicine and neuroscience. To date, only one of these has met all U.S. Food and Drug Administration requirements for use on humans. A component venom peptide of the piscivorous species *Conus magus*, now synthesized, has been commercially available since 2004 as a pain killer called Ziconotide or Prialt, a blocker of calcium channels in nerve cell membranes that carry pain signals to the spinal cord. The latter trade name is an acronym for “primary alternative,” referring to morphine, over which it has the advantage of being non-addictive [27]. Conniff recounts the recovery of a 60-year old lumber mill worker who suffered a crushed spine when he was rolled under a log in a sawmill. After three days of intrathecal injection of Prialt he was able to walk again “for the first time in years” [28].

### 2.8. Cone Snails as Murder Weapons in Fiction

A few murder mystery writers have ventured to fictionalize the dangers to humans from conotoxins and their delivery to inappropriate targets. The earliest I have been able to locate is the “The Cloth-of-Gold Murders,” by the well-known 20th century American mystery writer Baynard Kendrick (1894–1977), who published “The Cloth-of-Gold Murders” in the American Magazine’s then monthly feature, “Complete American Mystery Novel” in February 1958 [29]. A glance at Figure 2 shows that this time period was one of increasingly frequent media reports of human injuries and fatalities due to stings by *Conus*. Kendrick’s complex story of about 4000 words is subtitled “A killer prowled the peaceful island—his weapon a deadly, gleaming shell.” It involves two murders, a diverse cast including a malacologist and others with serious interests, and is set on the Florida Gulf Coast. The cast of *Conus* comprises two species, *C. textile* and *C. gloriamaris*, for whose exotic provenance the author provides a rational explanation [29].

A more recent (1986), small book-length mystery novel is also by a malacologically able author, Ann Kengalu, (d. 1998) who ventured to the Solomon Islands as an Anglican missionary and stayed to marry into a local family. It also involves murders and appearances of a couple of cone snails and cowries, but they have only cameo roles in the murders and are not weaponized. The book is “Murder on the Mataniko Bridge,” set and published in the Solomons and with a young woman from New Zealand as the central character [30].

Aside from print media, a 1972 episode of the television series *Hawaii 5-0* featured an apparent murder plan involving *Conus textile*, but instead resulted in the perpetrator’s suicide [31]. Yuhas also noted that “in the recent film *Jurassic Park 2* only cone snail venom was powerful enough to fell a *Tyrannosaurus rex*,” although the last dinosaurs had become extinct about seven million years before the first *Conus* appeared in the fossil record [32].

## 3. Discussion

The number of injuries inflicted by stings of humans by *Conus* can never be accurately determined, because many never have and never will be reported. Nevertheless, for the past 62 years the author has maintained a database of all cases for which he could locate reports in news media or medical records. Now updated and made electronically accessible, this database currently lists 141 cases during the period 1670–2017, caused by members of 34 *Conus* species. It includes 36 fatal cases and provides a sample adequate for determining (1) the species group within the hyperdiverse genus *Conus* that is most likely to envenomate humans and with the highest probability of fatality, and (2) the status and trends of these infrequent but potentially dangerous, primarily tropical interactions between man and molluscs.

Analyses of these data indicate that almost all, if not all, *Conus* whose envenomations have caused human deaths are of the single species *C. geographus*. This is a widely distributed, exclusively fish-hunting Indo-West Pacific species that occurs mainly on coral reefs from the intertidal zone to about 20 m. It is the largest piscivorous *Conus* species, also consistent with its being the most dangerous to humans.

The also large and widely distributed mollusc-hunting species *Conus textile* has been accused in print, especially prior to the 20th century, of a number of human fatalities. However, to my knowledge, none of these have been verified with additional confirmatory information. In addition, no other molluscivorous species, nor any piscivores other than *C. geographus*, have been demonstrated to cause human fatality.

Finally, one of the currently most promising and rapidly advancing aspects of *Conus* toxicology studies is the roles and evolutionary histories of the remarkably large numbers of distinct neurotoxic conopeptides produced by all of the hundreds of distinct *Conus* species. In particular, recent studies have revealed distinct offensively and defensively purposed conopeptides, and their disparate secretion sites in different regions of the venom gland (offensive venom peptides are produced and released mainly in the distal part of the venom gland, and their defensive counterparts, mainly in the proximal portion, relative to the venom bulb [26,27,33]. Studies of future cases of human injuries from envenomation by *Conus* that focus on collecting and analyzing perpetrator specimens, especially in addition to *C. geographus*, would enable broader tests of their venom glands for the presence of specific conopeptides that could be distinguished as having predatory versus defensive functions, proximal versus distal regionalized secretion areas of the venom gland, and similar versus disparate patterns of evolutionary histories in the diverse feeding guilds of this hyperdiverse genus.

## 4. Materials and Methods

The methods are essentially those of the prior compilation, updated and expanded to incorporate new analyses of data additional to the supplemental materials of that study [3]. For example, these have revealed significant temporal trends in the severity of human injuries, likely reflecting improved communication, transportation, knowledge, and access to improved medical care in remote tropical areas, especially during the past century.

Because the previously published tabulated data remain accessible as electronic supplemental material (SM1–SM9) to Ref. [3], they are here updated to include the more recent reports and are available at www.clinpharmacol.com, Vol. 54, issue 7. This website, maintained by the International Journal of Clinical Pharmacology and Therapeutics, is referred to here as the IJCPT site, and the updated online supplemental material to Ref. [3], as SM1–SM9.

## Figures and Tables

**Table 1:** Temporal patterns of fatal and non-fatal cases of human injuries due to *Conus* envenomations, ca. 1670–2017, by 25-year intervals (except first two and last entries).

Interval (Years)	Fatal in Interval	Non-Fatal in Interval	Total in Interval	% Fatal in Interval	Cumulative % Fatal	Species Added in Interval
1670–1699	1	0	1	100	100	1
1700–1849	0	1	1	0	50	1
1850–1874	2	2	4	50	50	1
1875–1899	1	3	4	25	40	2
1900–1924	1	1	2	50	42	0
1925–1949	7	4	11	64	52	1
1950–1974	15	51	66	23	30	17
1975–1999	8	36	44	18	26	9
2000–2017	0	8	8	0	25	2
Totals	35	106	141		25	34
